# Exosomes as a therapeutic tool to promote neurorestoration and cognitive function in neurological conditions: Achieve two ends with a single effort

**DOI:** 10.1111/cns.14752

**Published:** 2024-05-22

**Authors:** Solmaz Fallahi, Hamid Soltani Zangbar, Fereshteh Farajdokht, Reza Rahbarghazi, Gisou Mohaddes, Fariba Ghiasi

**Affiliations:** ^1^ Drug Applied Research Center Tabriz University of Medical Sciences Tabriz Iran; ^2^ Department of Physiology Tabriz University of Medical Sciences Tabriz Iran; ^3^ Department of Neuroscience and Cognition, Faculty of Advanced Medical Sciences Tabriz University of Medical Sciences Tabriz Iran; ^4^ Neurosciences Research Center Tabriz University of Medical Sciences Tabriz Iran; ^5^ Department of Applied Cell Sciences, Faculty of Advanced Medical Sciences Tabriz University of Medical Sciences Tabriz Iran; ^6^ Department of Biomedical Education California Health Sciences University, College of Osteopathic Medicine Clovis California USA

**Keywords:** cognition, exosome therapy, neurological disorders, neuroregeneration, neurorestoration

## Abstract

Exosomes possess a significant role in intercellular communications. In the nervous system, various neural cells release exosomes that not only own a role in intercellular communications but also eliminate the waste of cells, maintain the myelin sheath, facilitate neurogenesis, and specifically assist in normal cognitive function. In neurological conditions including Parkinson's disease (PD), Alzheimer's disease (AD), traumatic brain injury (TBI), and stroke, exosomal cargo like miRNAs take part in the sequela of conditions and serve as a diagnostic tool of neurological disorders, too. Exosomes are not only a diagnostic tool but also their inhibition or administration from various sources like mesenchymal stem cells and serum, which have shown a worthy potential to treat multiple neurological disorders. In addition to neurodegenerative manifestations, cognitive deficiencies are an integral part of neurological diseases, and applying exosomes in improving both aspects of these diseases has been promising. This review discusses the status of exosome therapy in improving neurorestorative and cognitive function following neurological disease.

## Graphical abstract

Neurological diseases (including Alzheimer's disease, Parkinson's disease, stroke, multiple sclerosis, epilepsy, traumatic brain injury, encephalopathy, etc.) lead to an increase in inflammation, oxidative stress and cell death in the brain, but exosome therapy reverses the process by reducing inflammation and improving neurogenesis, and ultimately leads to cognitive function improvement.

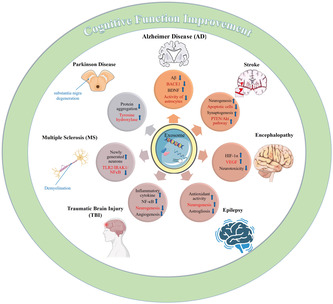

## 1 INTRODUCTION

Exosomes are nanosized (40–100 nm) membrane microvesicles that exist in almost all biological fluids, and there has been increasing attention to them over recent years.^1^ These microvesicles contain lipids, proteins, nucleic acids, mRNAs, non‐coding RNAs (ncRNAs), cytokines, and other bioactive substances.^2^ In recent investigations, the characterization of exosomal cargo through release and uptake has had a vital role in identifying their function in the nervous system.^3^ Many findings have demonstrated that exosomal cargo is essential in normal CNS communications, neural regeneration, synaptic function, and plasticity.^4^ Moreover, exosomes have some valuable characteristics that make them a potent player in treating various neurological conditions through experimental studies.^5^ Considering the small size of exosomes, they can credibly avoid macrophage polarization and effortlessly cross the extracellular matrix and biological barriers.^6^ As an advantage, surface CD55 and CD59 in exosomes inhibit coagulation factors and phagocytosis by suppressing some extracellular compounds, such as opsonin bound, and eventually distribute broadly in body fluids.^7^, ^8^

Due to the natural origin of exosomes, they have suitable biocompatibility, little immunogenicity, and benefit from many advantages in contrast to synthetic nanodelivery systems and liposomes.^9^, ^10^ Moreover, exosomes efficaciously impress target cells and overcome obstacles, such as the blood–brain barrier (BBB), to serve as a therapeutic choice and a potent natural intermediate for drug delivery.^11^, ^12^, ^13^ Various experimental studies have shown the amazing effects of exosome therapy in attenuating different neurological disease signs.^14^, ^15^ Exosomes have active participation in the nervous system function, and several types of neural cells, such as neurons, microglia, astrocytes, and oligodendrocytes, direct intercellular communications by secreting exosomes.^16^, ^17^, ^18^ Generally, exosomes of neural cells are involved in normal neurodevelopment, neuroregeneration, and synaptic function regulation.^19^, ^20^

During various neurological disorders, parallel with the change of exosomal content, the cognitive function of affected people is damaged in multiple manners; hence, exosomes are used as a suitable biomarker for diagnosing these diseases besides cognitive assessments.^21^, ^22^, ^23^ On the other hand, the administration of exosomes in various models of neurological diseases and, in some cases, inhibiting their pathologic release, besides improving neurogenesis in the central nervous system, has improved their cognitive performance in different dimensions, such as spatial memory and recognition.^24^, ^25^, ^26^ Given that, following neurological disorders such as stroke and Alzheimer's disease, despite neurodegenerative manifestations, cognitive functions like spatial memory and recognition are affected; therefore, different therapeutic approaches should target their cognitive function along with neurorestoration.^27^, ^28^, ^29^, ^30^, ^31^ Herein, we present the current state of exosome therapy in improving neurorestoration and cognitive function following neurological disease.

## 2 EXOSOMES CHARACTERISTICS AND BIOGENESIS

Various types of extracellular vesicles (EVs) have been identified to date, including apoptotic bodies (about 1 μm), ectosomes that consist of microparticles, microvesicles, shedding vesicles (about 100 nm–1 μm), and finally exosomes, that are specified with a 40–100 nm size.^32^ Exosomes have an endocytic origin with molecular ingredients, making them highly conserved among most eukaryotic organisms.^33^ Multiple characterization measures are needed to specify exosomes. International Society for Extracellular Vesicles (ISEV) presents that exosomes harvested from various biological fluids should be evaluated and confirmed by two types of proteins, like endosomal transmembrane proteins and recovered cytosolic proteins of EVs.^34^ Multiple evaluation procedures are used for measuring exosomes, including dynamic light scattering (DLS), nanoparticle tracking analyses (NTA), transmission electron microscopy (TEM), resistive pulse sensing (RPS), ELISA, flow cytometry, microfluidics and electrochemical detection.^35^

**FIGURE 1:**
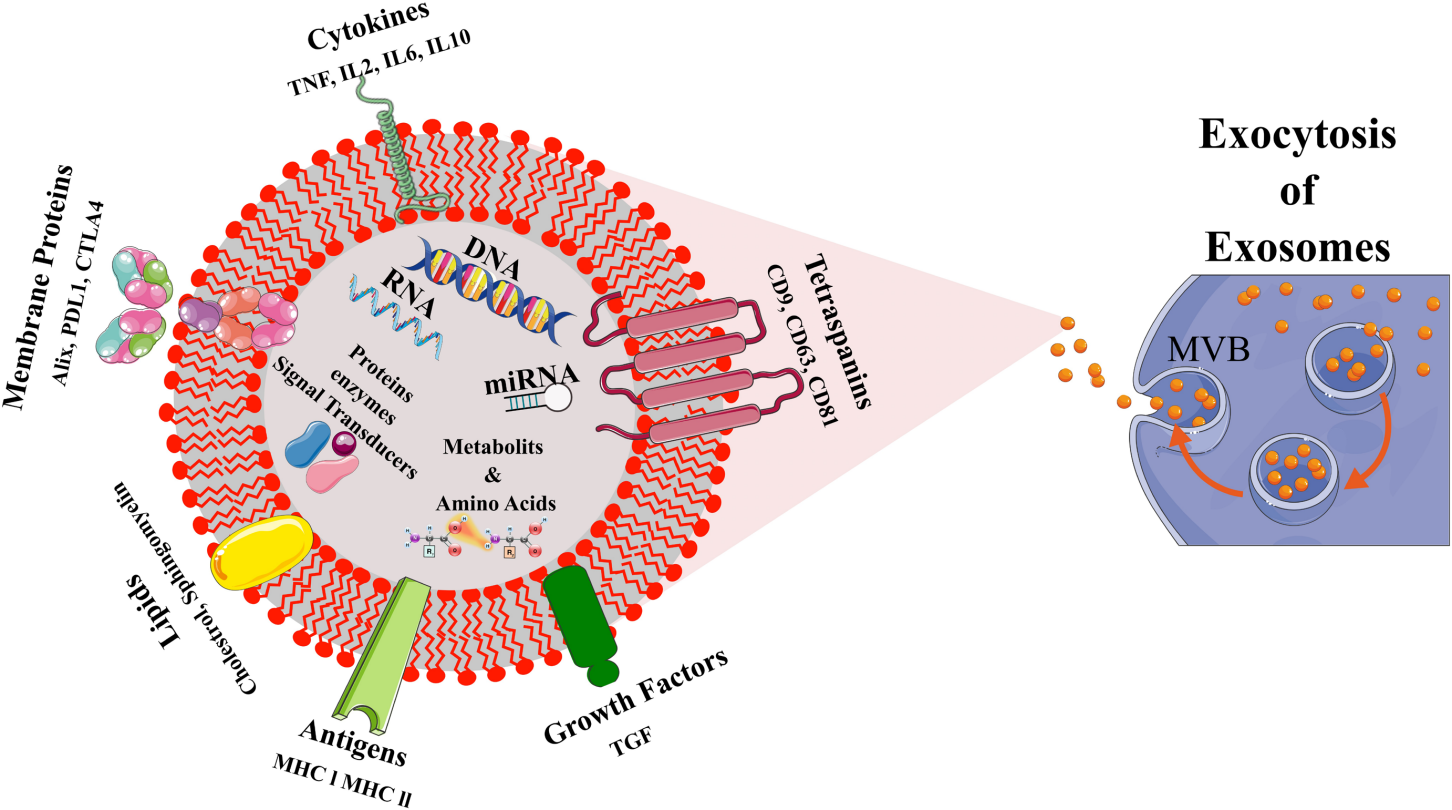
Exosome structure and composition. Exosomes are released to extracellular space following MVB fusion with the plasma membrane. Exosomes are nanosized compositions generally including nucleic acids, proteins, membrane tetraspins and proteins, Antigens, Growth factors, and lipids dependent on their origin and activation state. CTLA4, Cytotoxic lymphocyte antigen 4; IL, Interleukin; MHC, Major histocompatibility; MVB, Multi Vesicular Body; PDL1, Programmed Death‐Ligand 1; TGF, Transforming growth factor.

Biogenesis supplies the exosomes with nucleic acids, lipids, and proteins.^36^ Exosomes embrace multiple nucleic acids like DNAs, mRNAs and non‐coding RNAs like miRNAs.^37^ As a cargo content, lipids equip exosomes with cholesterol, sphingomyelin, desaturated lipids, and phosphatidylserine.^38^ Also, exosomes contain membrane or cytoplasmic proteins that consist of MVBs tetraspin proteins (such as CD9, CD81, CD63), major histocompatibility complex class I and II (MHC‐I, MHC‐II) antigens, enzymes, fusion proteins (PDL1, CTLA4, Alix), cytokines (IL2, IL6, IL10, TNF) growth factors (like TGF), and chaperones (Figure 1).^39^, ^40^, ^41^, ^42^

**FIGURE 2:**
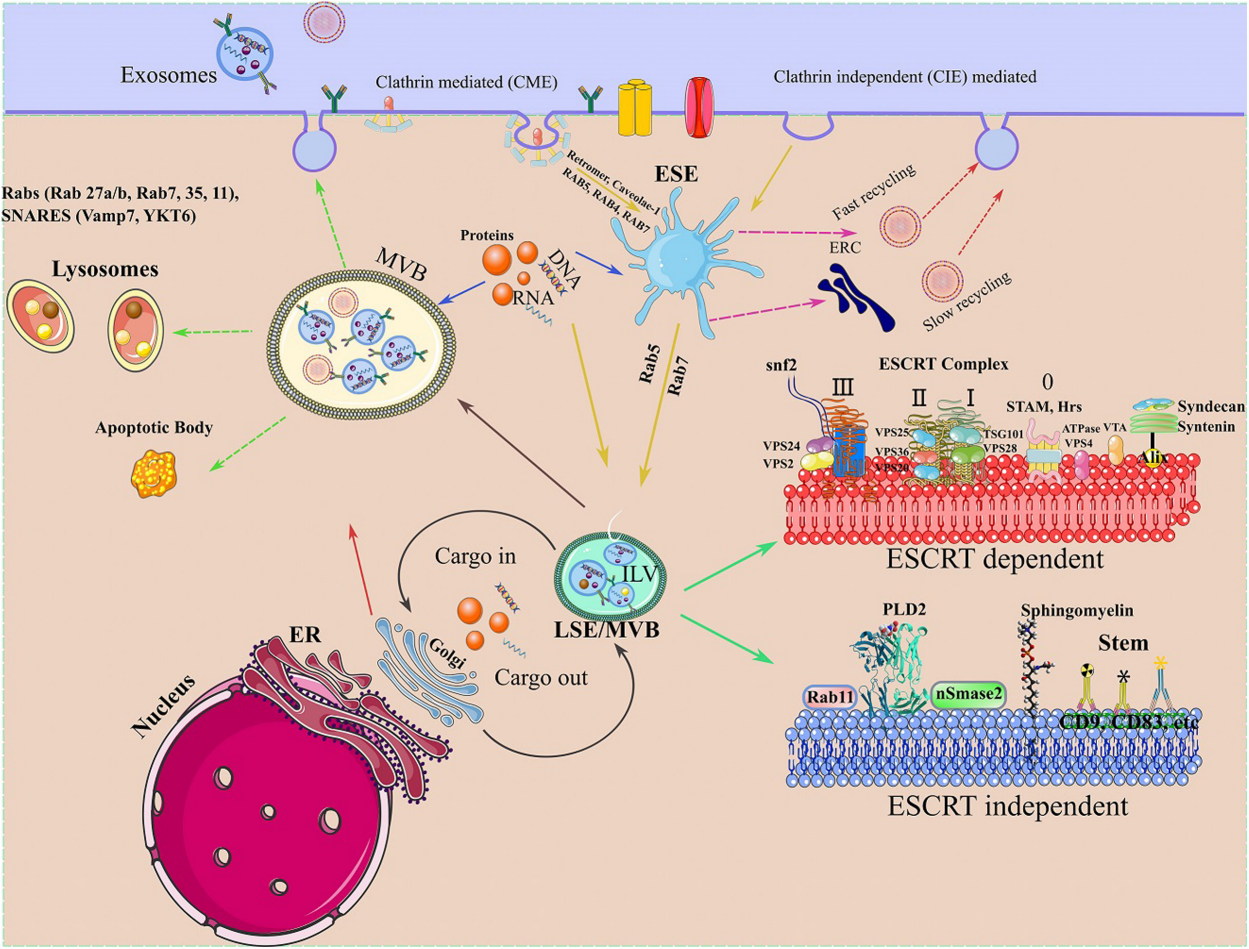
Mechanism of exosome biogenesis and secretion. Exosomes in the form of ILVs bud inwardly to create early endosomes and MVBs. The membrane invaginate by clathrinid‐mediated or independent endocytosis. Following endosome sorting, a portion of it recycles and goes back to the membrane quickly or slowly to recuperate vesicles (fast or slow recycling). The other section extends into multivesicular bodies (MVB) through the participation of some proteins, including Rabs (4, 5, 7), which pursue intracellular cargo transport and form the ILVs. The biogenesis of ILVs has two pathways: The ESCRT dependent and ESCRT‐independent. The ESCRT‐dependent pathway involves the ESCRT complex‐related portions and the “Syndecan–Syntenin–ALIX pathway.” The ESCRT independent pathway consists of some membranous lipids and tetraspanins, forming a domain as a base for cargo transition. Next to the MVB formation that contains numerous ILVs, it fuses with the lysosome or membrane. Some RAB proteins, such as RAB27a/b, RAB11, RAB7, RAB35, and some SNAREs, including Vamp7 and YKT6, participate in the fusion process of MVB.

Exosomes are formed from cytoplasmic membrane invagination that creates early sorting endosomes (ESE) and endosomes. The endosome buds inwardly and captures specific cytoplasmic molecules that lead to the creation of intraluminal vesicles (ILVs) (Figure 2).^36^ The ESE is a relatively “large vesicle” and the primitive membrane site, created by the merger of endocytic vesicles containing the tubular extension, which is developed by RAB5, RAB4, RAB7, RAB11, retromer, and caveolae‐1 molecules.^43^ The endocytic vesicles are constructed by clathrin‐mediated endocytosis (CME) or clathrin‐independent (CIE) ways.^44^ A part of sorted endosomes may revert to the membrane via “Fast recycling” or “Slow recycling” to recuperate vesicles.^45^ Eventually, multivesicular bodies (MVBs) or late‐sorting endosomes (LSE), that consist of ILVs, are transferred and unifies with the membrane.^46^ In the fusion process of MVBs with the membrane, some Rab (Rab 27a/b, Rab7, 35, 11) and SNARE (Vamp7, YKT6) proteins participate.^47^ On the other hand, some MVBs may be transported to lysosomes for degeneration and apoptotic body formation.^48^ The biogenesis mode of ILVs is classified into two categories, endosomal sorting complexes required for transport (ESCRTs) dependent and independent pathways.^49^ The ESCRT includes ESCRT‐0, ESCRT‐I, ESCRT‐II, and ESCRT‐III subcomplexes.^50^ Besides these subcomplexes, “Syndecan–Syntenin–ALIX pathway” related proteins and some accessory proteins like Vps twenty associated 1 (VTA‐1) and AAA ATPase vacuolar protein‐associate sorting (VPS)4 participate in the ESCRT complex.^51^, ^52^ ESCRT‐0 has two subunits, as ubiquitin‐binding domains (UBDs), including signal transducing adaptor molecule (STAM) and hepatocyte growth factor‐regulated tyrosine kinase substrate (Hrs), that help to identify ubiquitinated proteins of endosomal membrane and its internalization.^53^ A Hrs recruited clathrin coat further stabilizes the ESCRT‐0 subdomains and prevents the diffusion of the exosomal cargo.^54^ ESCRT‐0 and ESCRT‐I, by clathrin assistance and constructing a subdomain of the endosomal membrane, invaginates the ILV.^55^ TSG101, as a portion of ESCRT‐I, activates ESCRT‐II through different stimuli, and consequently, ESCRT‐I and ESCRT‐II complex create buds through confining and stabilizing the bud neck.^56^ ESCRT‐II attaches to the VPS28 component of ESCRT‐I via VPS36, continuing with the interaction of ESCRT‐II‐VPS25 and VPS20 subunits that recruit the ESCRT‐III to the endosome membrane.^57^ ESCRT‐II triggers the gathering of the ESCRT‐III complex. Subsequently, VPS24 and VPS2 construct ESCRT‐III complex following Snf7 homo‐oligomerization and contribute to finding an ESCRT‐III component containing vesicle‐shaping properties.^58^ After splitting the buds, the ESCRT‐III subdomain and AAA ATPaseVPS4 enroll enzymes to deubiquitinate tag from the cargo proteins before launching them into the ILVs.^59^

The ESCRT‐independent pathway is mediated via some mechanisms that include (1) Tetraspanin‐enriched microdomains (TEMs) that serve as cargo transporters upon interacting with intermediates such as CD83, CD9,^60^ (2) Overexpression of PLD2 that work as an effector to control the MVBs budding and exosomal development,^61^ (3) Activity of Rab11, and sphingomyelinase (nSMase, a ceramide‐producing enzyme).^62^

## 3 BLOOD–BRAIN BARRIER PERMEABILITY TO EXOSOMES

Barriers comprising the blood–brain barrier (BBB) and the choroid plexus protect the brain from attacking the peripheral agents and are considered the chief barriers of the brain regarding surface and length.^63^ Despite the importance of BBB in protecting the brain from exogenous threats and maintaining its homeostasis, the penetration of pharmacological agents such as exosomes through drug delivery approaches is vital in treating neuropsychological conditions.^64^ In the next part, following the explanation of BBB structure, we will clarify the mechanism of multiple agents, like exosomes, crossing from the BBB.

### 3.1 Structure of BBB

**FIGURE 3:**
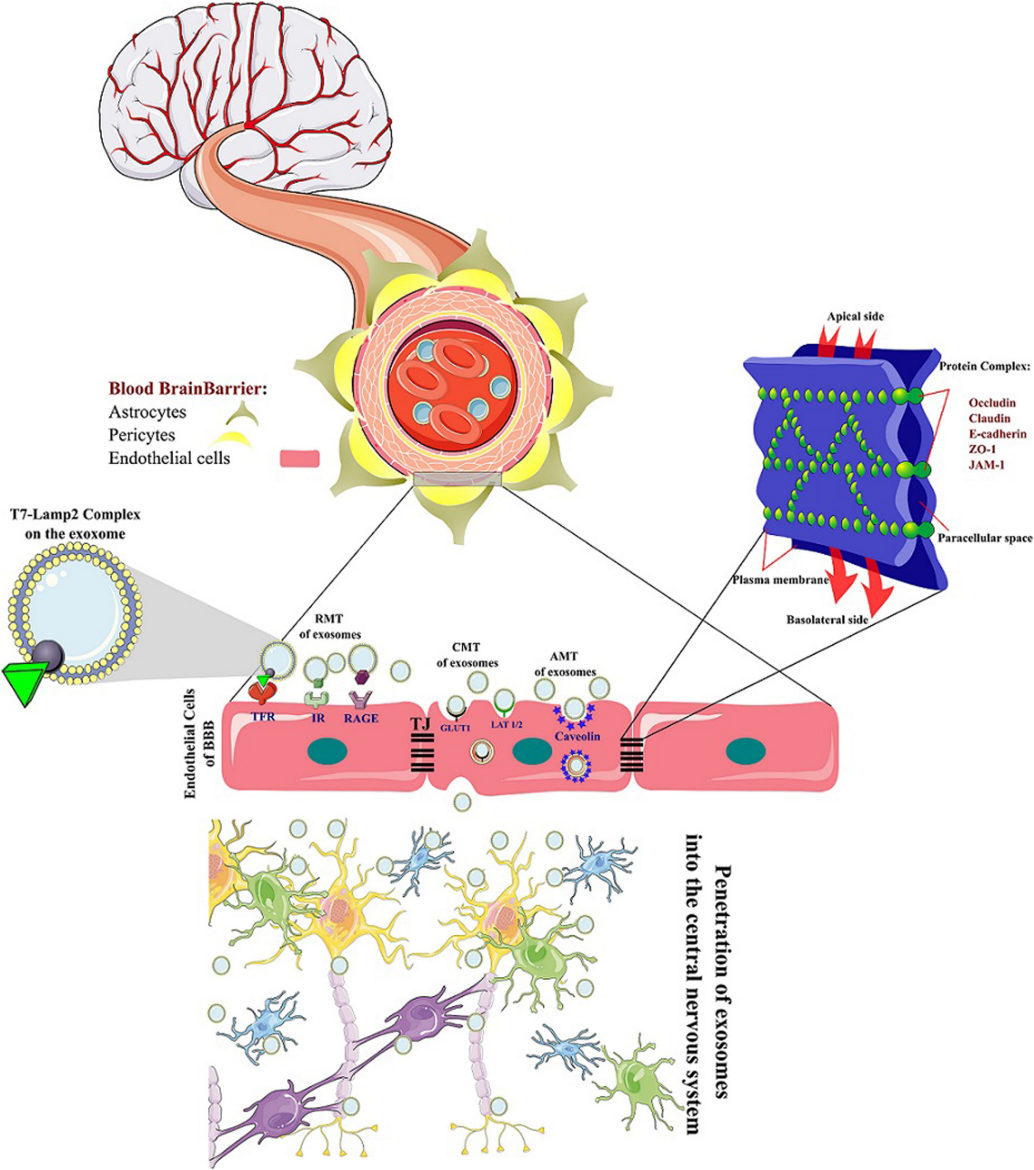
Structure of the BBB and mechanisms of exosomes cross from the BBB. BBB consists of endothelial cells (ECs), pericytes, astrocytes, and neuronal terminations. Tight Junctions (TJ), the major player of BBB structural integrity, include some major proteins such as claudin, occludin, Junctional adhesion molecules (JAMs), Zonula Occulin (ZO), and E‐cadherin. Exosomes can transport across the BBB with carrier‐mediated transport (CMT) through carriers such as GLUT 1 and LAT1/2. Another transport method is receptor‐mediated transport (RMT) through receptors such as TFR, IR, and RAGE. Also, Adsorptive‐mediated transport (AMT) is a receptor‐independent endocytosis that induces inward membrane curvature through oligomerization.

BBB comprises vessels assembled with endothelial cells (ECs), pericytes, astrocytes, and neuronal terminations.^65^ End feet of astrocytes are expanded along the basal lamina and form a narrow barrier. Pericytes fill the perivascular space between the astrocyte's end‐feet and capillary wall and operate vasculature tone, angiogenesis, repair, and constancy.^66^ Eventually, neuronal terminations, as part of the structure, reach all the cells that form the BBB. The tight junctions (TJ) and the adherens junctions (AJ) are the main subcellular responsible for BBB structural integrity. TJs seal the ECs to create a stable tubular construction and contain three major transmembrane proteins: (1) claudin, solely posited in TJ with four trans‐membrane segments containing two intracytoplasmic termini and two extracellular loops, (2) occludin, a protein with four transmembrane segments and three intracytoplasmic regions, and (3) junctional adhesion molecules (JAMs), as vital players in the conservation of TJ integrity and the junctional compound owing to their function in the maintenance of the cells together. Also, zonula occludens (ZOs) proteins play the role of scaffolding that provides a structural basis for TJs via the assembling of multiprotein complexes at the cytoplasmic surface of TJs (Figure 3).^67^ The astrocyte's end feet connect closely to ECs in a netlike manner and participate in creation, maintenance, and integrity of BBB.^68^

### 3.2 Mechanisms of exosome transmission from the BBB

Exosomes enter the host cell through variant manners, the most important of which include: (1) triggering a signaling cascade with conjunction to the G‐coupled receptor of the cell surface such as TFR, IR, and RAGE, and transporting exosomes (Receptor‐Mediated Transport/RMT); (2) fusion with the cell and releasing the cargo to the cytoplasm of the cell to induce the target cell signaling, that is mediated via oligomerization of caveolins and absorbing exosomes (adsorptive‐mediated transport/AMT); and (3) transcytosis with conjunction to the carriers of the recipient cell such as GLUT 1 and LAT1/2, that leads to endocytosis and storage of exosomes in the MVB (carrier mediated transport/CMT) (Figure 3).^69^, ^70^, ^71^ Exosomes must accede to ECs to pass through them and cross the BBB to enter the brain. During this mutual interaction, physical contact (fusion) and transcytosis facilitate the transmission of the exosome or its cargo through the BBB.^72^ Exosomes attach to ECs during fusion and release the cargo into the host cell's cytosol. Following cellular entry and transcytosis, depending on the size and density, exosomes degrade or are directed to endosomes and transferred to the abluminal surface of the ECs.^73^ Also, modification of the exosome surface facilitates their delivery and cross from the BBB to enter the brain and reach the target location. Hijacking RMT is a common strategy for transporting therapeutic agents and passing over the BBB.^74^ This strategy is efficient for exosome delivery by labeling target membrane peptides. T7 peptide, a TfR‐binding peptide, and the HAIYPRH sequence have been used as target peptides for exosome delivery (T7‐Exo). Joining the T7 peptide to Lamp2b and intravenous injection of this conjugation could attack the intracranial glioblastoma compared to unmodified exosomes.^75^

In several experimental studies, neurotropic virus‐derived peptides, such as RVG, induce brain targeting of exosomes. In a study, RVG expression at the exosomal membrane promoted its fusion with Lamp2b, an exosomal membrane protein, and improved brain delivery of siRNA‐loaded exosomes.^76^ Since the exact mechanism of the BBB crossing pathway of exosomes has not been shown, modified exosomes have promising and efficient delivery of siRNA to brain neural cells in experimental models.

## 4 EXOSOMES IMPROVE NEUROGENESIS IN THE BRAIN

Brain‐derived exosomes (BDEs) released by all cell types in the CNS contain cargo from the lineages that generate them. In physiological conditions, they are attractive as a vital mediator of cellular communications and waste control between neural cells, glial cells, and the brain's connective tissue.^77^ Hippocampal neurons secrete exosomes that participate in cargo transfer into other neurons and facilitate activity‐dependent translation.^78^ In the denervated hippocampal niche like fimbria–fornix transection (FFT) condition, the proliferation and neurogenesis of neural stem cells (NSCs) improve in the subgranular zone (SGZ).^79^ RNA sequencing analysis from the extracted hippocampal exosomes in the FFT rat model demonstrated that miR‐3559‐3P and miR‐6324 increase after FFT. Transfection of these miRNAs' mimics inhibits NSCs' proliferation but promotes differentiation.

**FIGURE 4:**
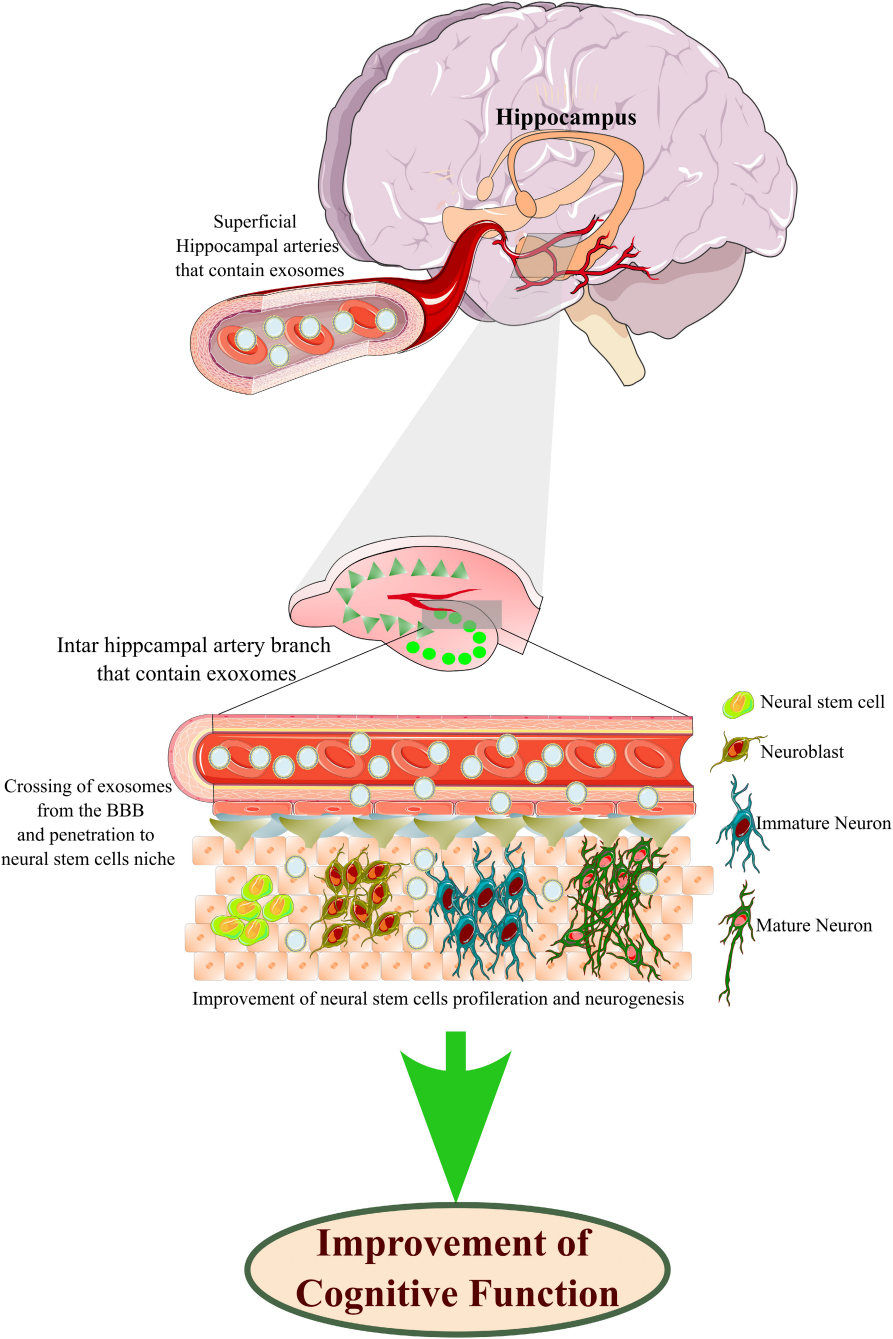
Importance of exosomes in the improvement of hippocampal neurogenesis. Recent findings implicate the effective role of exosome delivery in the neurogenic niches of the brain, including the hippocampus, so it promotes the generation of granular cells from neuroblasts in the dentate gyrus. Exosomes that may home in arteries, such as the inter‐hippocampal artery, after administering, enter the central nervous system and neurogenic niches that finally pass through the blood–brain barrier and improve cognitive function by enhancing neurogenesis.

On the other hand, inhibitors of miR‐3559‐3p and miR‐6324 promote NSCs proliferation and inhibit their differentiation to neurons.^80^ Considering the critical role of exosomes in neural communications of the brain, recent findings implicate the influential role of exosome therapy in the neurogenic niches of the brain, including the hippocampus, so that it promotes the generation of granule cells from neuroblasts in the dentate gyrus and subsequently improves cognitive function^81^ (Figure 4). By administration of exosomes derived from human‐induced pluripotent stem cells (hiPSCs) into the rodents brain, exosomes incorporate into the soma of cells in the DG of the hippocampus, which leads to higher proliferation of neural stem cells (Ki‐67^+^ cells) and high density of DCX^+^/ BrdU^+^ cells in the DG.^82^ In the next section, we will discuss the effect of exosome delivery on improving cognitive function and neurorestoration in different neurological disorders.

## 5 EXOSOME THERAPY ENHANCES NEURORESTORATION, CONCURRING WITH COGNITIVE FUNCTION IMPROVEMENT IN NEUROLOGICAL DISORDERS

Previous findings have confirmed that stem cells interpose their therapeutic consequence in neurological conditions by paracrine mechanisms. Among the paracrine products of stem cells, exosomes are specially used to treat various neurological diseases by carrying the paracrine factors to the degenerated site and promoting regeneration. The effective therapeutic potential of derived exosomes from different cell lines like neural stem cells, mesenchymal stem cells, and embryonic stem cells have been observed in neurological conditions by promoting cognitive function, neurological recovery, neurogenesis, regeneration, synaptic plasticity, immunomodulation, and tissue repair (Table 1).

**TABLE 1:** The role of exosome therapy in various neurological conditions.

Neurological condition	Origin of exosome	Mechanism of neurorestoration	Effect on cognition	Task	Indexes	References
Alzheimer	HUMSCs in 3D cells culture	↓ Aβ	↑ Spatial learning and memory	MWM	↓ Escape latency	^30^
		↑ α‐secretase			↑ Distance & Time in target Quadrant	
		↓ β‐secretase				
	Engineered exosomes containing miR‐29	↓ BACE1	↑ Spatial memory	Barnes maze	↓ Escape latency	^74^
		↓ BCL2‐like 11			↑ Target seeking & Goal hole exploration	
	Exosomes isolated from hippocampal NSC (NSC‐exo)	↓ Amyloid beta oligomers (Aβo)	↑ Recognition	NORT	↑ Spent time with the novel object	^75^
		↑ Long‐term potentiation (LTP)				
	Human brain microvascular endothelial cells (HBMVECs) derived exosomes	↑ P‐gp	↑ Spatial memory	MWM	↑ Time in the target quadrant	^76^
		↓ AΒ			↓ Escape latency	
	Mesenchymal stem cell‐derived exosomes (MSC‐exos)	↑ Uptake of 18F‐ FDG	↑ Recognition	NORT	↑ Frequency of tendency to the central area	^78^
		↓ AΒ	↑ Recognition	OFT	↑ Rearing & Traveled distance in the central part	^81^
		↑ Sphingosine kinase/sphingosine‐1‐phosphate signaling pathway		NORT	↑ Preference index of the NORT	
		↓ Hyperactivity of hippocampal microglia and astrocytes	↑ Spatial memory and Recognition	MWM	↑ Swimming time in the target quadrant	^82^
		↓ IL‐1β, IL‐6, TNF‐α		NORT	↑ Discrimination index in the NORT	
		↑ AΒ1‐42, p‐Tau BDNF				
		↑ PSA‐NCAM and DCX in the sub‐ventricular zone (SVZ)	↑ Spatial memory	MWM	↑ Time in the target quadrant	^83^
					↓ Escape latency	
	Exosomes derived from human umbilical cord mesenchymal stem cells	↓ AΒ accumulation and Neuroinflammation in Microglial activity	↑ Spatial memory	MWM	↑ Time in the target quadrant	^79^
					↓ Escape latency	
	Exosomes derived from hypoxia‐preconditioned mesenchymal cells	↓ Synaptic dysfunction	↑ Spatial memory	MWM	↑ Distance & Spent time in the target quadrant	^80^
		↑ IL‐4 and IL‐10				
		↓ TNF‐α and IL‐1β STAT3				
		↓ NF‐κB				
	Exosomes harvested from curcumin‐primed cells (Exo‐cur)	↑ AKT/GSK‐3β pathway	↑ Spatial memory	MWM	↓ Escape latency	^84^
		↓ Phosphorylation of the Tau			↑ Number of crossing	
					↑ Target quadrant occupancy indexes	
	Plasma exosomes loaded with Quercetin (Exo‐Que)	↓ Cyclin‐dependent kinase 5 (CDK5)‐mediated phosphorylation of Tau	↑ Spatial memory	MWM	↓ Escape latency	^85^
					↑ Number of crossings	
	RVG‐tagged MSC‐Exo	↓ AΒ levels and plaque deposition	↑ Spatial memory	MWM	↓ Escape latency	^86^
					↑ Spent time in the target quadrant	
	Exosomes derived from the biomimetic silibinin‐loaded macrophage	↓ AΒ aggregation	↑ Spatial memory	MWM	↓ Escape latency	^87^
		↓ Activity of astrocytes			↑ Spent time in the target quadrant & Crossing time	
Parkinson's Disease (PD)	EVs derived from human teeth stem cells	↑ Tyrosine hydroxylase (TH)	↑ Spatial learning & memory	MWM	↑ Spent time in the target quadrant	^93^
					↓ Escape latency	
	Microglia‐derived exosomes containing α‐synuclein	↑ Protein aggregation	No significant effect on spontaneous alternation, as an index of spatial memory, of injured animals in the Y‐Maze	Y‐maze	Spontaneous alternation	^94^
Multiple Sclerosis	Intravenous administration EVs	↓ Brain atrophy	↑ Passive avoidance memory	Passive avoidance	↓ Latency to enter the dark compartment	^99^
		↑ Neural stem cells proliferation in the SVZ				
		↓ Inflammatory cytokines				
	MSCs harvested exosomes	↑ Numbers of newly generated neurons	↑ Social Recognition	Social behavior test	↑ Spent time in the column that stranger	^100^
		↓ TLR2/IRAK1/NFκB pathway				
Stroke	Exosomes derived from CCR2 receptor‐overexpressing HUC‐MSCs	↑ Bind to CCL2	↑ Spatial memory	MWM	↓ Escape latency	^102^
		↑ Remyelination & Oligodendrogenesis			↑ Spent time in the target quadrant	
		↑ Macrophage polarization				
	NSC EV	↑ M2 cells	↑ Non‐episodic memory	Tail suspension	↓ Immobile state	103, 104
		↓ Th 17		NORT	↑ Spent time with the novel object	
	Transmission of exosomal microRNA‐124 from neurons to microglia	↑ CX3CL1/CX3CR1 pathway	↑ Spatial memory	MWM	↓ Escape latency	^105^
	Exosomes derived from human umbilical cord blood derived CD133+ cells (CD133+Exo)	↑ Synaptogenesis	↑ Spatial memory and Recognition	NORT	↑ Discrimination index	^106^
		↑ White matter remodeling		MWM	↓ Escape latency	
	Serum exosomes	↑ The ratio of Bcl‐2 / Bax	↑ Recognition	OFT	↓ Traveled distance	^107^
		↓ Apoptotic cells			↓ Spent time in the central part of the OF	
		↓ Cleaved caspase‐3				
	Serum exosomes of young rats	↑ CD46, as a C3b/C4b inactivator	↑ Spatial learning and Long‐term memory	MWM	↓ Latency	^108^
		↓ Iba1			↑ Traveled distance to find the platform	
	Extracellular vesicles from adipose‐derived stem cells (ADSC‐EVs)	↑ Polarization of microglia type2 (M2)	↑ Spatial working memory	T‐Maze Step through test	↑ The rate of alternation	^111^
		↑ Repair and Proliferation of endothelial cells			↑ Time spent in the dark zone & number of dark zone entrance	
	BMSC‐Exosomes	↓ NLRP3 inflammasome & pyroptosis relevant proteins in neurons	↑ Spatial memory	MWM	↓ Escape latency	^112^
					↑ Spent time in the target quadrant	
	EVs derived from adipose tissue stem cells (hAT‐MSC)	↑ PTEN/Akt pathway	↑ Spatial memory	MWM	↓ Latency	^14^
		↑ Angiogenesis	↑ Working memory	NORT	↑ Time on the novel object	^114^
		↑ Regulating protein transduction	↑ Short & Long‐term memory	Y‐Maze	↑ Spontaneous alternation	
			↑ Anxiety‐like Behaviors	EPM	↑ Spent time in the open arm	
				OFT		
	Zeb2/Axin2‐supplemented exosomes	↑ Neurogenesis	↑ Spatial memory	MWM	↓ latency to find the platform	^31^
Encephalopathy	Human amniotic fluid‐derived exosomes (hAFEXOs)	↑ HIF‐1α	↑ Spatial memory	MWM	↓ Escape latency	^121^
		↑ VEGF			↑ Spent time in the target quadrant	
Epilepsy	MSCs derived exosomes	Nrf2‐NF‐KB signaling pathway	↑ Spatial memory	MWM	↓ Escape latency	^125^
		↓ Activity of A1 astrocytes			↑ Spent time in the target quadrant	
	MSC‐EVs are enriched in antioxidant miRNAs	↑ Nrf2 system & Renovation of hippocampal neurons	↑ Spatial memory	MWM	↓ Escape latency	^126^
		↑ Antioxidant activity			↑ Spent time in the target quadrant	
	Intranasal administration of A1‐exosomes derived from human bone marrow MSCs	↓ Neuron loss & Inflammation	↑ Recognition	OLT	↑ Exploring the novel place object	^23^
		↑ Neurogenesis		NORT	↑ Affinity for novel object	
	IL‐1‐Exo that isolated from IL‐1‐treated MSCs	↓ Astrogliosis & inflammatory reaction of astrocytes in the lipopolysaccharide (LPS)	↑ Spatial memory	MWM	↑ Spent time in the target quadrant	^128^
					↓ Escape latency	
Traumatic brain injury (TBI)	Exosomes derived from multi pluripotent MSCs	↓ Inflammation	↑ Spatial memory	MWM	↓ Escape latency	^132^
		↑ Neurogenesis & Endogenous angiogenesis			↑ Spent time in the target quadrant	
	Human umbilical cord mesenchymal stem cells (HUCMSCs) derived exosomes	↓ Inflammatory cytokine production	↑ Spatial memory	MWM	↓ Escape latency	^133^
		↓ NF‐κB signaling pathway			↑ Spent time in the target quadrant	
		↓ Neuronal apoptosis				
		↑ Cortical neural regeneration				
	Exosomes derived from MSCs cultured under 2D conventional and 3D collagen scaffolds conditions	↓ Neuroinflammation	↑ Spatial memory	MWM	↓ Escape latency	^134^
		↑ Generation of newborn endothelial cells & newborn mature neurons			↑ Spent time in the target quadrant	
	miR‐17‐92 cluster‐enriched exosomes derived from MSCs	↑ Angiogenesis & Neurogenesis	↑ Spatial learning and memory	MWM	↑ Spent time in the target quadrant	^109^
		↓ Neuroinflammation			↑ Cross time	
	Human MSC derived exosomes (MSCexo)	↑ Hippocampal neural cell loss & Neurogenesis & Angiogenesis	↑ Spatial memory	MWM	↓ Escape latency	^110^
		↓ Inflammation			↑ Spent time in the target quadrant	
	Microglial exosomes	↑ MiR‐124‐3p Rela/ApoE signaling Pathway	↑ Recognitive	NORT	↑ Spent time with the novel object	^113^
			↑ Spatial memory	MWM	↑ Spent time in the target quadrant	
	Exosome that derived from cortical astrocytes and transfected with shRNA, Bcl‐2, Bax	↓ Hippocampal apoptosis	↑ Spatial memory	MWM	↑ Spent time in the target quadrant	^14^
		↑ Hippocampal EPSP amplitude				
Diabetes‐induced cognitive impairment	Intracranial injection of MSCs derived exosomes	↓ Synaptic loss & the degeneration of hippocampal neurons	↑ Spatial learning & memory	MWM	↑ Spent more time in the target quadrant	29, 139
	Enriched environment enhances exosomal miR‐146a secretion from bone BM‐MSCs	↓ Expression of IRAK1, NF‐κB	↑ Spatial learning & memory	MWM	↑ Spent time in the target quadrant	^140^
		↓ Tumor necrosis factor‐α			↓ Escape latency	
	Loading miR‐146a in to the BECDEs and implementation of this content into the ventricle of diabetic mice	↓ PrPc levels	↑ Short‐term memory	NORT	↑ Discriminatory index	^142^
				Y‐Maze	↑ Spontaneous alternation	
	BECDEs	↓ Evolving neurovascular dysfunction	↑ Recognition	Odor recognition tests	↑ Discriminatory index	^129^
			↑ Spatial memory	MWM	↑ Spent time in the target quadrant	

### 5.1 Alzheimer's disease (AD)

The function of exosomes and their content, including miRNAs and mRNAs, alters in AD.^83^ As a neurological condition, AD downregulates the expression level of some mRNAs, like Chi3l1, since it up‐regulates the expression level of some mRNAs, like Rhog.^84^ The misexpression of amyloid precursor protein (APP) is crucial in driving neuropathological cascades, leading to AD, and isolated exosomes from an animal's brain with AD promote APP's overexpression in neuronal N2a cells. Besides, harvested exosomes from N2a cells with abnormal APP expression dysregulate APP expression in host normal N2a cells, mediated by the low expression of exosomal miR‐185‐5p.^85^ Generally, exosomal miRNAs are effective biomarkers for predicting AD a few years before the onset of cognitive malfunctions.^84^ As exosomes and their cargo help to diagnose this disease, in recent years, exosome therapy to treat AD and improve the cognitive function of sufferers has been considered by researchers.^86^ 3D cell culture‐derived exosomes from human umbilical cord mesenchymal stem cells (hUMSCs) up‐regulate the expression of α‐secretase and down‐regulate the β‐secretase and diminish the production of Aβ in both pathological cell lines and transgenic mice model of AD through their special cargo.^25^ Injection of engineered exosomes containing miR‐29 to the CA1 region of the AD rats downregulates the expression of BACE1 (β‐site amyloid precursor protein cleaving enzyme 1) and BIM [Bcl − 2 interacting mediator of cell death (BCL2‐like 11)], and show a significant premiere exploration for the goal sector (GS) of the Barnes maze.^87^

**FIGURE 5:**
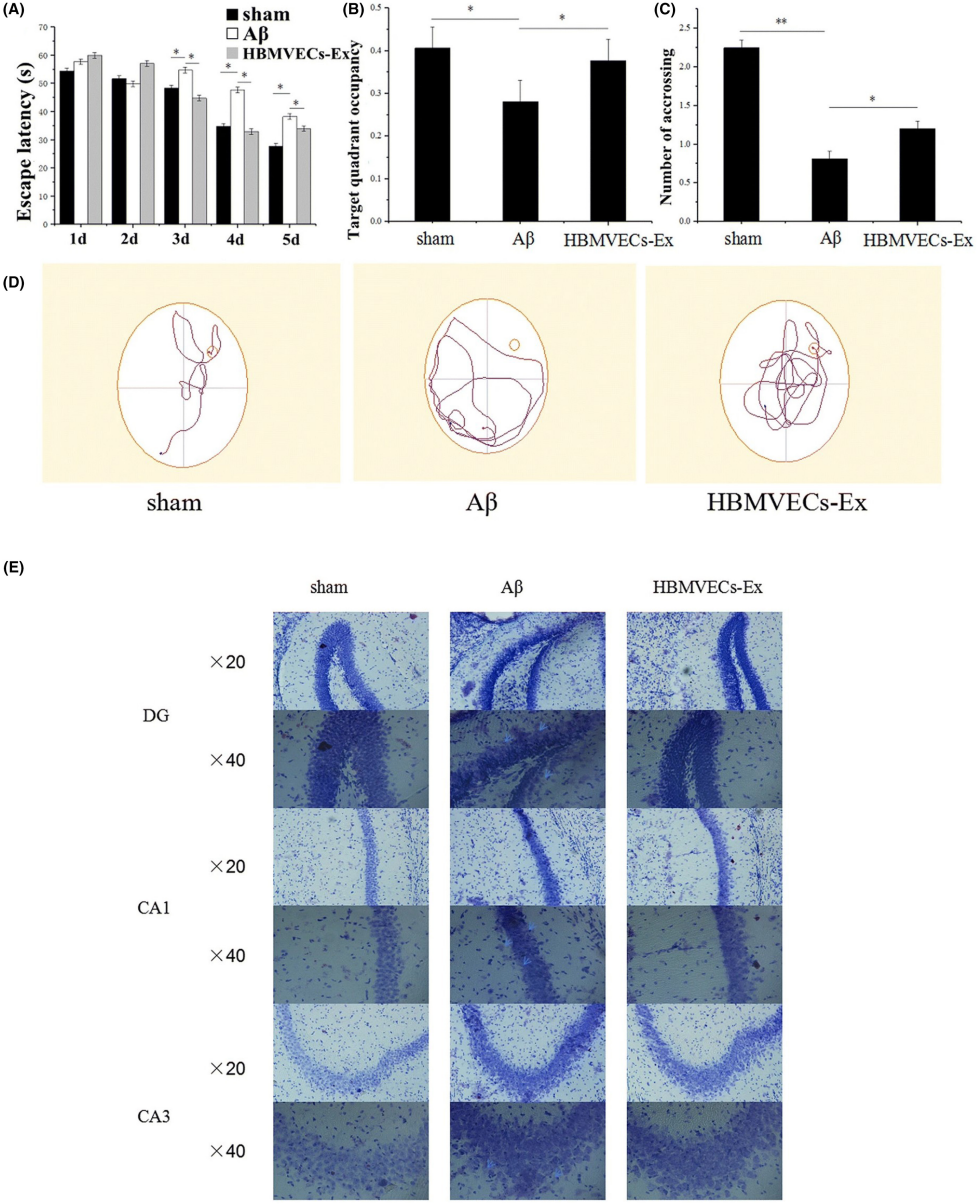
Brain microvascular endothelial cell‐derived exosomes (HBMVECs‐Ex) potently improve cognitive function by enhancing the clearance of Aβ in mouse model of AD. (A‐D) Decrease of escape latency, increase of target quadrant occupancy, and increase of platform crossing number in the MWM after treating animals with HBMVECs‐Exs. (E) Representative Nissl staining of different hippocampal regions. Reproduced with permission.

Exosomes isolated from hippocampal neural stem cells (NSC‐Exo) protect the hippocampus from amyloid beta oligomers (Aβο)‐induced suppression of long‐term potentiation (LTP) and improve the cognitive function of animals, confirmed by Novel Object Recognition (NORT) test. In this study, impaired animals subjected to Aβο and treated with NSC‐Exo significantly spent more time exploring the novel object through the choice phase of the NORT.^88^ Human brain microvascular endothelial cells (HBMVECs)‐derived exosomes, containing P‐glycoprotein (P‐GP), enhance the clearance of Aβ by distinguishing capture between Aβ and P‐GP, and considering the results of Morris Water Maze (MWM) task, improve the spatial memory of animals given escape latency and spent time in the target quadrant of the maze (Figure 5).^89^ Although mesenchymal stem cell‐derived exosomes (MSC‐exos) are an emerging and admirable therapeutic approach for AD.^90^ MSC‐exosomes enhance the uptake of [18F]FDG in the brain and improve the function of AD animals in the NORT test, like NSC‐Exo.^91^ Exosomes derived from human umbilical cord mesenchymal stem cells (hucMSC‐exosomes) ameliorate Aβ accumulation and neuroinflammation in AD animals by modulating microglial activity and also considering the Morris Water Maze (MWM) task; these exosomes improve the function of animals in the escape latency and spent time in the target quadrant of the maze.^92^ Exosomes derived from hypoxia‐preconditioned MSCs have a similar outcome on the function of APP/PS1 mice in the MWM by alleviating synaptic dysfunction, up‐regulating anti‐inflammatory cytokines (IL‐4 and ‐10), down‐regulating proinflammatory cytokines (TNF‐α and IL‐1β), and reducing the activity of STAT3 and NF‐κB in the brain.^93^ Bone marrow mesenchymal stem cells‐derived exosomes (BMSC‐Exo) improve cognitive function via decreasing Aβ accumulation and animating the sphingosine kinase/sphingosine‐1‐phosphate pathway.^94^ Administration of these exosomes to the lateral ventricle in the streptozotocin (STZ) model of AD inhibits the hyperactivity of hippocampal microglia and astrocytes, downregulates the expression level of IL‐1β, IL‐6, TNF‐α, Aβ1‐42, p‐Tau and up‐regulate the expression of BDNF as an essential synapse‐related protein. This neural restoration outcome positively correlates with increased frequency of tendency to the central area of the open field test (OFT), increased rearing, and traveled distance in the central part of the OFT and the preference index of the NORT.^95^ In a study by Edvin and colleagues, they investigated the effect of MSC‐Exo on the cognitive recovery and neurogenesis in the rodent model of AD, which was established by bilateral injection of beta‐amyloid 1–42 aggregates into the DG of the hippocampus. MSC‐Exo therapy improved the expression of PSA‐NCAM and DCX in AD animals' sub‐ventricular zone (SVZ). Also, they had more swimming time in the target quadrant of MWM and a better discrimination index in the NORT.^96^ Exosomes harvested from curcumin‐primed cells (Exo‐cur) prevent neural death through in vitro and in vivo conditions and activate the AKT/GSK‐3β pathway that ameliorates the cognitive dysfunction of animals by inhibiting phosphorylation of the Tau. In the probe trial of MWM, after removing the platform, Exo‐cur treated animals had higher target quadrant occupancy and increased numbers of crossing the platform place compared to AD animals.^97^ Also, plasma exosomes loaded with Quercetin (Exo‐Que) relieve the cognitive symptoms of okadaic acid (OA)‐induced AD by inhibiting cyclin‐dependent kinase 5 (CDK5)‐mediated phosphorylation of Tau. AD animals that were treated with Exo‐Que had improvement in the escape latency, the number of crossings, and target quadrant occupancy indexes of MWM,^98^ as well as, RVG‐tagged MSC‐Exo improves the targeting of exosomes to the hippocampus and cerebral cortex after intravenous administration, therefore sharply decrease Aβ levels and plaque deposition parallel with a decrease in the escape latency time, and increase of platform location crossing in the MWM.^99^ Exosomes derived from the biomimetic silibinin‐loaded macrophage have the same effect on the function of an animal in the MWM through inhibition of Aβ aggregation and activity of astrocytes.^100^

### 5.2 Parkinson's disease (PD)

Exosomes' content alters after PD and is a promising potential target for biomarker development in PD.^101^ A positive correlation exists between the exosomal content like plasma L1CAM, exosomal Linc‐POU3F3 levels, and nonmotor symptoms of PD patients like mood and attention/memory.^102^ In PD, the plasma exosomal prion concentration negatively correlates with the cognitive function level assessed with the Montreal Cognitive Assessment (MoCA).^103^ Meanwhile, exosomes serve as a delivery vehicle via loading small interfering RNAs or proteins, allowing site‐specific targeting through the PD treatment.^104^

Upon transport of lung‐derived exosomes, the localized transcription factors of exosomes regulate the gene expression at the substantia nigra, and superior frontal gyrus regions of PD.^105^ EVs derived from the human teeth stem cells successfully normalize the expression of tyrosine hydroxylase (TH) in the substantia nigra (SN) and striatum of 6‐OHDA‐induced PD in rats and reverse the impairment of spatial learning/memory performance, such that the escape latency to find the platform in MWM decrease, and spent time in the target quadrant increase after the treatment.^106^ Microglia as a primary phagocyte of the nervous system, and their released exosomes can influence α‐synuclein pathology, but injecting microglia‐derived exosomes containing α‐synuclein, despite inducing protein aggregation in the recipient neurons, had no significant effect on spontaneous alternation, as an index of spatial memory, of injured animals in the Y‐Maze.^107^

### 5.3 Multiple Sclerosis

Multiple sclerosis (MS) is a major demyelinating disease that exhibits demyelination in the CNS concomitantly with neurological deficits and cognitive impairments.^108^ Common treatments for MS consist of immunosuppressors and preventives of brain immune infiltrations.^109^ However, these therapeutic approaches do little to enhance myelin renovation besides harmful side effects. Instead, as naturally occurring small vesicles, exosome therapy promotes remyelination and neurorestoration in MS by delivering mRNA and other exosomal cargo.^110^

Serum‐derived exosomes that contain mir‐219 and are generated by young rats significantly reduce oxidative stress and increase the hippocampus's oligodendrocyte precursor cell levels and myelin content in the lysolecithin‐induced in vitro MS Model.^111^ Intravenous administration of EVs minimizes brain atrophy, increases neural stem cell proliferation in the SVZ, and decreases inflammatory cytokines levels in the serum of mice infected with Theiler's murine encephalomyelitis virus (TMEV), a progressive model of MS. Also, EVs attenuate motor deficits and improve passive avoidance memory (learning to avoid a noninvasive foot shock) of infected animals by encouraging remyelination and diminishing brain atrophy.^112^ In Experimental autoimmune encephalomyelitis (EAE) and cuprizone (CPZ) diet models of MS, MSCs harvested exosomes cross the BBB and significantly increase the numbers of newly generated neurons, inhibit the TLR2/IRAK1/NFκB pathway, and improve the function of animals in the social behavior test, such that after the MSC‐Exo treatment, animals of CPZ model spent more time in the column that stranger (unfamiliar) animal is in there.^113^

### 5.4 Stroke

**FIGURE 6:**
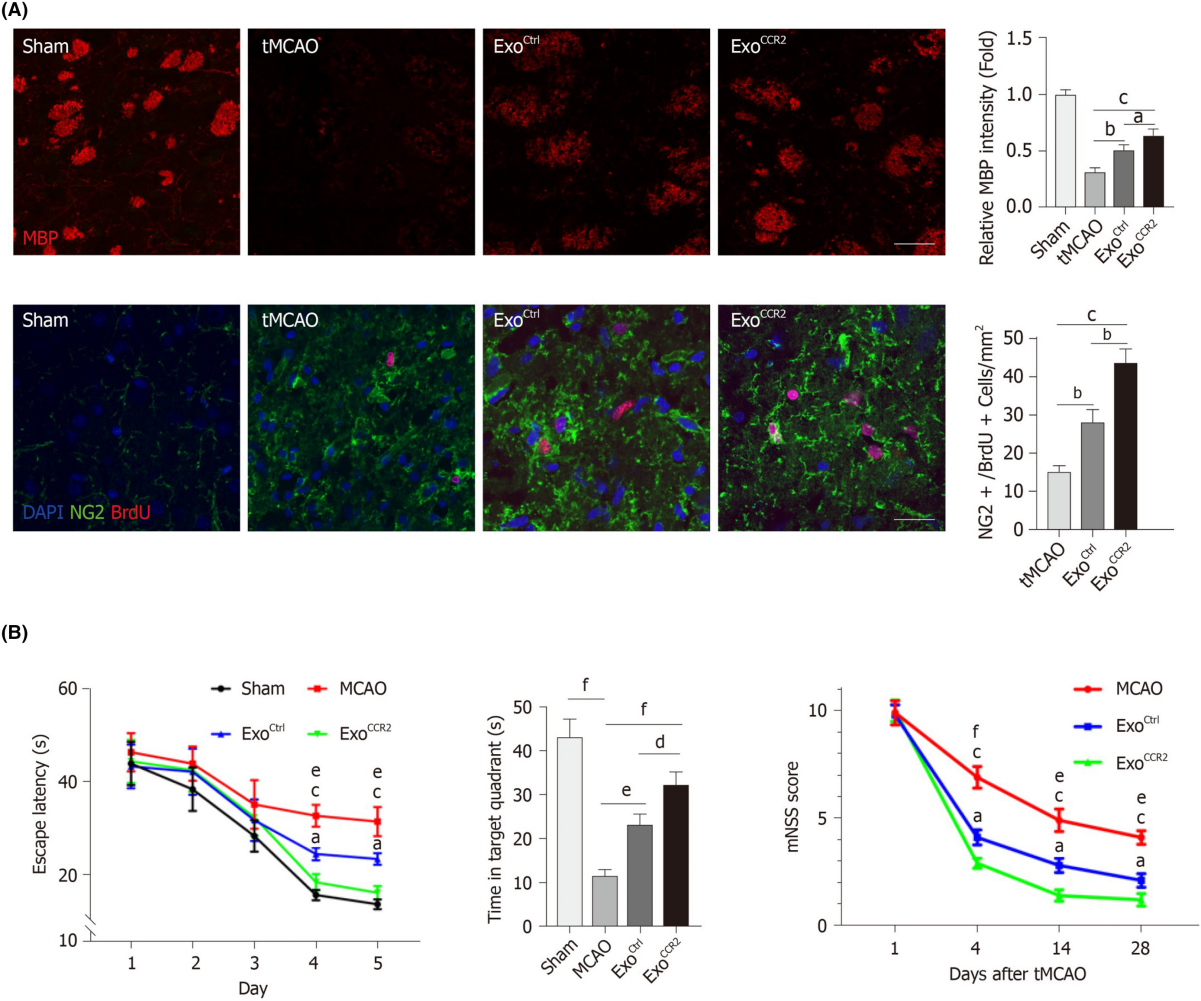
Beneficial effects of exosome therapy on the neurorestoration and cognitive function after the stroke. (A) Exosomes derived from CCR2 receptor‐overexpressing HUC‐MSCs improve the intensity of myelin binding protein (MBP), indicating the integrity of myelin, and also increase the number of BrdU+/NG2+ cells, indicating the oligodendrocyte proliferation around the ischemic area of animals with middle cerebral artery occlusion (MCAO). (B) These Exosomes have increased mean escape latency and the spent time in the target quadrant of MWM, in animals with MCAO. Treated animals had better motor function than untreated animals based on mNSS values. Reproduced with permission.

Exosomes from different cell lines reduce inflammation, enhance neurogenesis, and improve cognitive function after stroke.^14^ In a study by Yang and colleagues, exosomes derived from CCR2 receptor‐overexpressing HUC‐MSCs, significantly bind to CCL2 and have a dramatically beneficial effect on the remyelination, oligodendrogenesis, macrophage polarization, and cognitive function of post‐stroke cognitive impairment (PSCI) model (Figure 6).^114^ Neural stem cell extracellular vesicles (NSC EV) treatment in a rodent model of MCAO positively improves motor function and strength through beam walk and hanging wire tests and increases the spent time with the novel object in NORT, indicating the improvement of the episodic memory formation.^115^ Furthermore, NSC EVs improve non‐spatial memory in the murine thromboembolic stroke model evaluated by NORT, where the discrimination index showed that MCAO animals have a significant cognitive deficiency. Still, NSC EV‐treated animals had significantly better cognitive function than the injured group and, as a result, spent more time with the novel object compared to the familiar object in the NORT.^115^ One of the reasons for these improvements may be due to the CX3CL1/CX3CR1 pathway, which triggers a cascade and attenuates early brain injury (EBI) after subarachnoid hemorrhage (SAH) via promoting the transmission of exosomal microRNA‐124 from neurons to microglia, which in turn improves the escape latency of animals during the MWM as a spatial memory test.^116^

There is a significant correlation between measures of cognitive tests, such as discrimination index in novel odor recognition test or escape latency in MWM with left ventricular ejection fraction, in type 2 diabetes mellitus (T2DM) stroke rodents that are treated with derived exosomes from human umbilical cord blood CD133+ cells (CD133 + Exo).^117^ Treatment with healthy serum exosomes protects the BBB by reversing autophagy‐induced reduction of TJs and remarkably decreases apoptotic cells in the striatum, increases the ratio of Bcl‐2 to Bax, and inhibits cleaved caspase‐3, which coincides with lower traveled distance and spent time in the central part of the OF.^118^ Also, serum exosomes of young rats are rich in CD46, as a C3b/C4b inactivator, and systemic administration of these exosomes from young rats into aged ischemic ones decreases the expression of Iba1 and improves spatial learning and long‐term memory, so in treated animals, the latency and traveled distance to find the MWM platform reduce significantly.^119^ In recent years, MSCs‐based secretomes have been potentially appealing approaches in treating ischemic stroke.^120^, ^121^ Extracellular vesicles from adipose‐derived stem cells (ADSC‐EVs) promote the polarization of microglia type2 (M2), resulting in increased repair and proliferation of endothelial cells in the peri‐ischemia of transient middle cerebral artery occlusion (tMCAO) animals. Also, ADSC‐EVs treatment after cerebral ischemia increases the alternation rate in the T‐Maze, the time spent in the dark zone of the step‐through test, and the number of dark zone entrances.^122^

BMSC‐Exosomes improve animals' spatial memory after the MCAO, followed by reperfusion, by downregulating the expression of the NLRP3 inflammasome and pyroptosis‐relevant proteins in neurons.^123^ After induction of subarachnoid hemorrhage in rats, BMSC‐exosomes alleviate early brain injury (EBI) through miRNA129‐5p‐HMGB1 Pathway,^124^ and transfer of miR‐21‐5p enriched exosomes from MSCs, besides significant amelioration of EBI, alleviate neural apoptosis, and improve spatial memory of injured animals via the PTEN/Akt pathway.^125^ EVs derived from adipose tissue stem cells (hAT‐MSC) of the healthy individual, referred to as liposuction, reverse the destructive effect of cerebral stroke on motor function, working memory, short‐term memory, long‐term memory, and anxiety‐like behaviors. EVs‐delivered animals spent more time with the novel object in the NORT, had more spontaneous alternation in the Y‐Maze, and spent more time in the open arm of the EPM.^126^ Furthermore, Zeb2/Axin2‐supplemented exosomes, harvested from BMSCs transfected plasmid, improve the endogenous neurogenesis and neurobehavioral recovery of ischemic animals, especially in spatial memory manner with decreasing the latency to find the platform in the probe trial of MWM test.^26^

### 5.5 Encephalopathy

Exosomes can play a role in treating encephalopathies and their diagnosis as a biomarker.^23^, ^127^ Chronic traumatic encephalopathy (CTE), a neurological condition with prior exposure to repetitive head impacts, is associated with tauopathy and higher exosomal Tau in serum and correlates with worse performance in psychomotor and memory tests.^128^ After the occurrence of CTE, inhibition of brain‐derived exosomes release, enhanced glucose uptake, altered cytokine production trends, and significantly reversed cognitive impairment, such that treated mice spent less time finding the platform during the MWM and spent more time with the novel object in the NORT.^129^ Also, in the Gut–microbiota–brain axis that relates the intestinal microbiota and sepsis‐associated encephalopathy (SAE), intestinal epithelial cell (IEC)‐derived exosomes induce M1 polarization and secretion of pro‐inflammation factors like IL‐1β M1 that impairs the cognitive function of rodents; however, GW4869 inhibits the secretion of exosomes and decrease the distance and latency time to find the platform in MWM test.^130^ Hypoxic encephalopathy triggers a kind of CNS dysfunction with high mortality and morbidity in neonates and even life‐lasting paralysis.^131^ Brain‐derived extracellular vesicles (BEVs) ameliorate the neurotoxicity in oxygen–glucose deprivation (OGD) brain slices based on a dose‐time dependent procedure.^132^ Human amniotic fluid‐derived exosomes significantly augment the expression level of hypoxia‐inducible factor 1 α (HIF‐1α) and vascular endothelial growth factor (VEGF) that coincide with a decrease in the escape latency time and increase in the target quadrant occupancy of MWM test.^133^

### 5.6 Epilepsy

Epilepsy, a neurological disorder, leads to an abnormal electrical discharge of neurons. Emerging findings have confirmed that exosomes could be released following epilepsy and serve as a biomarker for diagnosis.^134^, ^135^ Since inflammatory pathways and astrocyte malfunction are involved in the evolution of epilepsy, suppressing inflammatory pathways and astrocytes is a promising strategy in epilepsy treatment.^136^ It has been shown that MSCs‐derived exosomes attenuate the activity of A1 astrocytes in the animal model of temporal lobe epilepsy (TLE) by regulating the Nrf2‐NF‐KB signaling pathway.^137^ MSC‐EVs are enriched in antioxidant miRNAs and, following seizure damage, improve the spatial memory of animals through the MWM test via renovation of hippocampal neurons and remarkable antioxidant activity associated with the Nrf2 defense system.^138^ Intranasal administration of NSC‐EVs after seizure induction reduces the expression of inflammatory cytokines, including IFN‐γ, TNF‐⍺ and IL‐1β.^82^ Also, intranasal administration of A1‐exosomes derived from human bone marrow MSCs, incorporated into the hippocampal neurons and confine the progress of status epilepticus SE‐induced dysfunction into chronic hippocampal impairment, such that treated animals represent a greater tendency for exploring the novel place object (NPO) in object location test (OLT), and more affinity for novel object area (NOA) in NORT.^139^ IL‐1‐Exo isolated from IL‐1‐treated MSCs significantly inhibits the astrogliosis and inflammatory reaction of astrocytes in the lipopolysaccharide (LPS)‐induced model of SE, and simultaneously decreases the escape latency time, increases the spent time in the target quadrant, and the number of platform crossings in the MWM.^140^

### 5.7 Traumatic brain injury (TBI)

TBI is closely associated with neuroinflammation, neuropathological protein accumulation, and ectopic release and spread of brain‐derived exosomes (BDE). After TBI, the rate of phosphorylated Tau elevates remarkably in exosomes. TBI‐isolated exosomes enforce toxicity in neural cultures, aggravate LTP impairment, and exacerbate cognitive and motor deficits after TBI.^141^ Current studies have confirmed that cell‐based therapies in experimental and clinical research can substantially improve motor and cognitive recovery by reinforcing neurogenesis and neurite growth following TBI. Moreover, many studies have shown that exosomal miRNAs are leading therapeutic candidates for improving neurorestoration after the TBI.^142^ Inhibition of BDE release after repetitive mild traumatic brain injury (TBI) increases glucose uptake, decreases neuropathological protein accumulation, and reverses cognitive deficiency in injured mice. GW4869 (a nMase inhibitor), as an inhibitor of BDE, changes the production outline of cytokine and enhances microglial proliferation, parallel with a significant decrease in escape latency (MWM) and a significant increase in the spent time with the novel object in the NORT.^129^ Exosomes derived from multi‐pluripotent MSCs, improve sensory‐motor and cognitive recovery in rats after TBI by reducing inflammation and promoting neurogenesis or endogenous angiogenesis.^143^ In a study by Zhang and colleagues, human umbilical cord mesenchymal stem cells (HUCMSCs) derived exosomes significantly decreased inflammatory cytokine production by repressing the NF‐κB signaling pathway, inhibited neuronal apoptosis, promoted cortical neural regeneration, and improved cognitive function of animals after the induction of TBI in rats.^144^

After the TBI, systemic administration of exosomes derived from MSCs cultured under 2D conventional and 3D collagen scaffold conditions reduces neuroinflammation, increases newborn endothelial cells' generation in the boundary zone, and remarkably increases newborn mature neurons in the hippocampus. These changes led to the improvement of animal spatial memory performance in the modified MWM, such that with each training trial, the platform was randomly relocated within the target quadrant to act as a probe trial.^145^ Also, miR‐17‐92 cluster‐enriched exosomes derived from MSCs improve motor function based on the modified neurological severity scores (mNSS) test, and especially the spatial learning and memory based on the MWM test, so that exosome‐delivered animals spent more time in the target quadrant and passed the platform of the maze more times.^146^

Human MSC‐derived exosomes (MSCexo) significantly decrease hippocampal neural cell loss, enhance neurogenesis and angiogenesis, ameliorate inflammation, and improve spatial memory after the TBI in a dose–response and window–response manner, measured by MWM. In this study, treated animals spent more time in the target quadrant and had less latency in finding the platform.^147^ Furthermore, MSCexo enhances long‐term memory processing and recall, spatial memory, prioritization, and identification of color in the modified operant conditioning model.^148^ Intra‐nasal delivery of exosomes derived from adipose‐derived stem cells (hASCexo) decreases cortical damage after the TBI and improves animals' cognitive function in the reversal trial of 8 arms radial arm water maze.^149^

Like MSCs‐derived exosomes and GW4869, microglial exosomes with up‐regulated miR‐124‐3p improve the cognitive function of rmTBI animals in the NORT and MWM indicators via Rela/ApoE signaling pathway that promotes the breaking of β‐amyloid proteolytic and thereby inhibit β‐amyloid malformations.^150^ Exosomes that derived from cortical astrocytes and transfected with plasmids expressing short hairpin RNA (shRNA), B‐cell lymphoma‐2 (Bcl‐2), and Bcl‐2‐associated X‐protein (Bax), significantly attenuated hippocampal apoptosis, increased hippocampal EPSP amplitude and increased spent time in the target quadrant of MWM.^151^

### 5.8 Other diseases

#### 5.8.1 Psychiatric disorders induced cognitive impairment

Exosome applications are not restricted to neurological conditions; in recent years, they have also been used in experimental research to treat psychiatric disorders, which are accompanied by cognitive deficits.^152^, ^153^, ^154^ It has been shown that exosomes isolated from human umbilical cord MSC considerably enhance neural stem cell differentiation, improve rodent recognition memory, and mitigate stress‐related symptoms generated by intrahippocampal injection of streptozotocin (STZ).^155^

Guoa et al. found that exosome therapy enhanced the sucrose preference index and increased activity levels in depressed animals. Exosome treatment increased hippocampus neurogenesis and raised the expression of miR‐26a and superoxide dismutase (SOD) while simultaneously decreasing the expression of TNF‐α and IL‐1β.^156^ Intranasal delivery of MSC‐derived EVs in the phencyclidine (PCP) model of schizophrenia leads to amelioration of schizophrenia‐like behaviors, including improved social interaction and sensorimotor gating as measured by the three chambers social interaction test and the paradigm of prepulse inhibition of the acoustic startle response. This is achieved by significantly increasing the number of GABA‐producing neurons in the prefrontal cortex, a severely affected area in schizophrenia.^157^

#### 5.8.2 Diabetes‐induced cognitive impairment

Cognitive impairment due to diabetes is a global problem. Many studies have confirmed that cognitive dysfunctions are much more prevalent in diabetic patients than in non‐diabetic people.^158^ Understanding the exosomal cargo can help to diagnose and provide practical treatments for diabetic patients.^159^ Intracranial injection of MSCs‐derived exosomes reverses diabetes‐induced cognitive disorder.^160^ These exosomes improve synaptic loss, and the degeneration of hippocampal neurons in STZ‐diabetic mice, and exosome‐delivered animals spent more time in the target quadrant of the MWM test.^24^ Also, an enriched environment enhances exosomal miR‐146a secretion from bone BM‐MSCs, decreases scape latency, and increases the time spent in the target quadrant of MWM after diabetes‐induced cognitive impairment in rats.^161^ In type 2 diabetes mellitus (T2DM) mice, brain endothelial cell‐derived exosomes (BECDEs) increase miR126 expression in the brain and serum and significantly improve their cognitive function.^162^ Loading miR‐146a into the BECDEs and implementing this content into the ventricle of diabetic mice decrease PrPc levels and restore short‐term memory confirmed by NORT and Y‐Maze so that treated animals significantly have a better discriminatory index and spontaneous alternation.^163^ Besides, reduced evolving neurovascular dysfunction and MRI analysis exhibited that BECDEs treatment in type 2 diabetes mellitus (T2DM) animals show significant elevation of relaxation time constant T2 and cerebral blood flow (CBF) in the brain white matter and amplification of relaxation time constant T1 and reduction of BBB permeability in gray matter. BECDEs significantly increased T1 and CBF in the hippocampus of T2DM animals. Besides, CEC‐Exo reduced diabetes‐induced cognitive deficits, and treated animals spent more time in the target quadrant of MWM and recognized different odors in the odor recognition tests.^164^

#### 5.8.3 High fat diet (HFD) induced cognitive impairment

HFD, correlated with cognitive impairments and neurological deficits, increases the risk of neurological disorders like Alzheimer's disease later in life.^165^, ^166^ HFD inhibits CREB phosphorylation in the hippocampus and downregulates the expression of CREB target genes (Bdnf, nNOS, Sirt1, Egr3, and RelA genes). Still, intranasal administration of NSC‐derived exosomes epigenetically restores the transcription of these genes by the recruitment of CREB and improves recognition and spatial memory of animals analyzed with NORT and object place recognition (OPR) test.^167^

#### 5.8.4 Hypothermic circulatory arrest induced cognitive impairment

The neurologic deficit remains a significant complication after cardiovascular surgeries with deep hypothermic circulatory arrest (DHCA).^168^ Exosomes derived from gene‐modified MSCs, protect the brain against prolonged DHCA through overexpression of microRNA‐214 (miR‐214) and enhance spatial memory of injured animals, so as the latency time of MWM decreases and platform location crossing increases in treated animals.^169^

## 6 CONCLUSION

**FIGURE 7:**
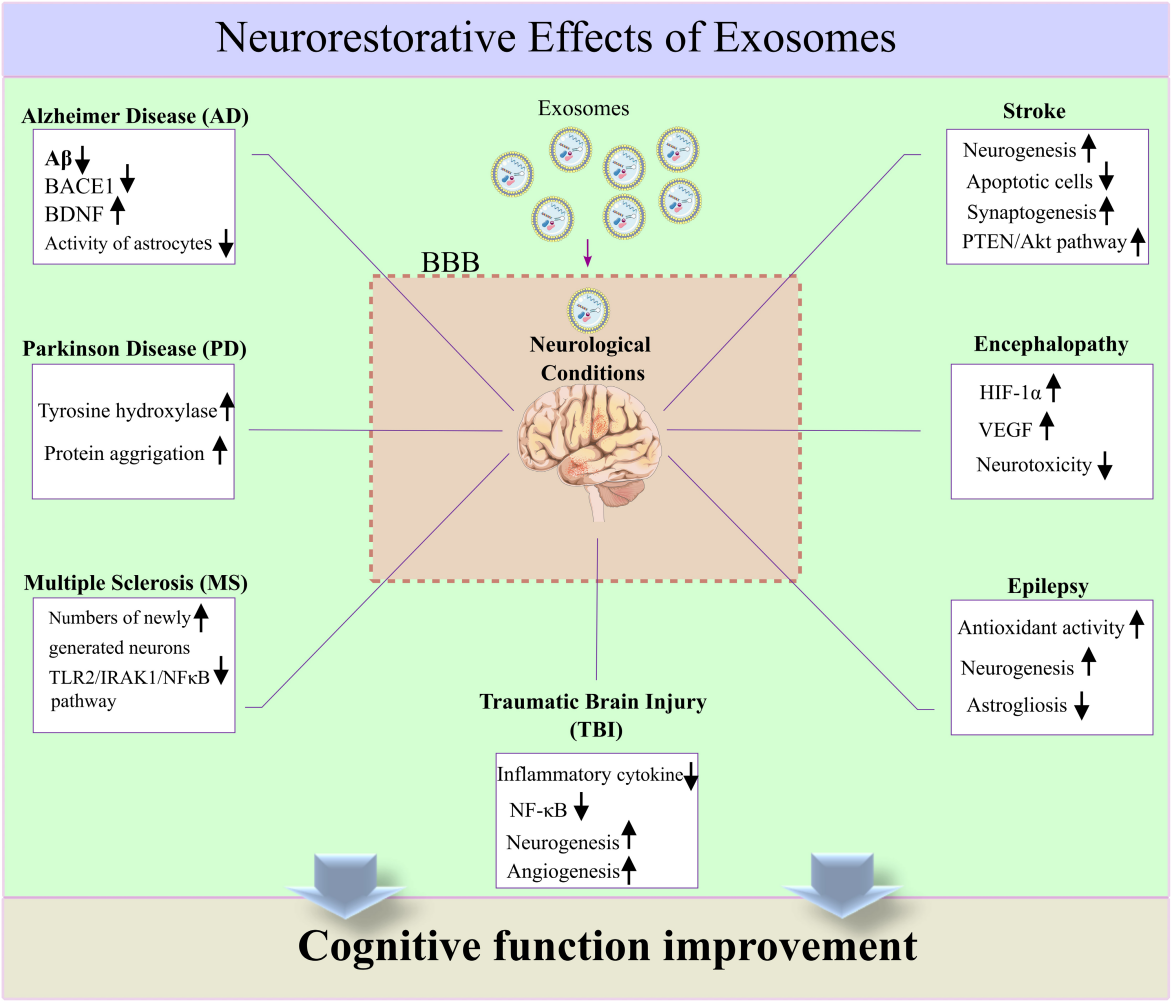
An overview of the beneficial effects of exosome therapy in neurological diseases. Exosomes have the advantage of low immunogenicity and simple cross from the BBB, enabling them to be a potent drug delivery vehicle that improves neurorestoration and cognitive function in neurological conditions by improving neurogenesis and decreasing neuroinflammation.

Exosomes are vital in cells' physiological functions and interactions. The use of exosome therapy in neurological diseases such as PD, AD, TBI, MS, stroke, epilepsy, encephalopathies, and other neurological conditions which affect cognitive function is developing (Figure 7). Implementing exosomes from multiple cell lines and sources has been shown to evoke neurorestorative effects in neurological models that accompany cognitive function recovery, too. Generally, compared to cell‐based therapy, exosome therapy has the advantage of low immunogenicity and simple cross from the BBB, enabling them to be a potent drug delivery vehicle that improves neurorestoration and cognitive function in neurological conditions. However, using these worthy cellular secretomes in humans remains challenging because of their safety and efficient application in the clinic, so many concerns still need to be solved, such as exosome optimal dose, timing, and other challenging problems.

## AUTHOR CONTRIBUTIONS

FGH, GM, HSZ, and SF designed the project and wrote the manuscript. SF and HS performed the animal modeling and experimental procedures. FF and RRGH helped with the data collection. SF and HS analyzed the data. FGH and GM provided funding acquisition. All authors have read and approved the final manuscript.

## FUNDING INFORMATION

This study was supported by a grant (67159) from Drug Applied Research Center, Tabriz University of Medical Sciences (Tabriz, Iran).

## CONFLICT OF INTEREST STATEMENT

The authors declared no Conflict of interest.

## Data Availability

All data generated or analyzed in this study are available from the corresponding author on reasonable request.
