# Correction to Exosomes as a therapeutic tool to promote neurorestoration and cognitive function in neurological conditions: Achieve two ends with a single effort. *CNS Neurosci Ther*. 2024 May;30(5):e14752. doi: 10.1111/cns.14752

**DOI:** 10.1111/cns.14844

**Published:** 2024-07-03

The authors affiliation list was updated.

In the author list, the affiliation address of Reza Rahbarghazi was changed to two institutional affiliations

Solmaz Fallahi^1,2^, Hamid Soltani Zangbar^3^, Fereshteh Farajdokht^1,2,4^, Reza Rahbarghazi^5,6^, Gisou Mohaddes^1,2,3,4,7^, Fariba Ghiasi^1,2^

^1^Drug Applied Research Center, Tabriz University of Medical Sciences, Tabriz, Iran

^2^Department of Physiology, Faculty of Medicine, Tabriz University of Medical Sciences, Tabriz, Iran

^3^Department of Neuroscience and Cognition, Faculty of Advanced Medical Sciences, Tabriz University of Medical Sciences, Tabriz, Iran

^4^Neurosciences Research Center, Tabriz University of Medical Sciences, Tabriz, Iran

^5^Stem Cell Research Center, Tabriz University of Medical Sciences, Tabriz, Iran

^6^Department of Applied Cell Sciences, Faculty of Advanced Medical Sciences, Tabriz University of Medical Sciences, Tabriz, Iran

^7^Department of Biomedical Education, California Health Sciences University, College of Osteopathic Medicine, Clovis, California, USA

We apologize for this error.

